# Improved CEEMDAN and PSO-SVR Modeling for Near-Infrared Noninvasive Glucose Detection

**DOI:** 10.1155/2016/8301962

**Published:** 2016-08-22

**Authors:** Xiaoli Li, Chengwei Li

**Affiliations:** School of Electrical Engineering and Automation, Harbin Institute of Technology, Harbin 150001, China

## Abstract

Diabetes is a serious threat to human health. Thus, research on noninvasive blood glucose detection has become crucial locally and abroad. Near-infrared transmission spectroscopy has important applications in noninvasive glucose detection. Extracting useful information and selecting appropriate modeling methods can improve the robustness and accuracy of models for predicting blood glucose concentrations. Therefore, an improved signal reconstruction and calibration modeling method is proposed in this study. On the basis of improved complete ensemble empirical mode decomposition with adaptive noise (CEEMDAN) and correlative coefficient, the sensitive intrinsic mode functions are selected to reconstruct spectroscopy signals for developing the calibration model using the support vector regression (SVR) method. The radial basis function kernel is selected for SVR, and three parameters, namely, insensitive loss coefficient ε, penalty parameter *C*, and width coefficient γ, are identified beforehand for the corresponding model. Particle swarm optimization (PSO) is employed to optimize the simultaneous selection of the three parameters. Results of the comparison experiments using PSO-SVR and partial least squares show that the proposed signal reconstitution method is feasible and can eliminate noise in spectroscopy signals. The prediction accuracy of model using PSO-SVR method is also found to be better than that of other methods for near-infrared noninvasive glucose detection.

## 1. Introduction

Diabetes is a chronic disease that poses a serious threat to human health. According to the International Diabetes Federation (IDF) in 2014, diabetes affects 387 million individuals around the world, and this figure is expected to increase to 592 million in 2035 [1]. The diabetes, cancer, and cardiovascular diseases are the main causes of death since 2005 [2]. At present, diabetes is treated by detecting blood glucose concentrations to adjust the dose of glucose-lowering drugs, thus controlling blood glucose levels to prevent and reduce the symptoms of diabetes and its complications [3]. The accurate detection of blood glucose concentrations is important in diabetes prevention and treatment.

Diabetes monitoring is usually carried out in hospitals or through self-monitoring [4]; diabetes monitoring commonly involves invasive detection, which uses high amounts of biochemical reagents, entails long testing times, and causes inevitable pain and inconvenience to patients. By contrast, noninvasive blood glucose detection [5–7] offers a number of advantages, such as fast analysis speed, absence of trauma, low cost, and environmental friendliness. Noninvasive optical detection technology [8–10] is an important research topic in the area of noninvasive blood glucose detection. Since the 1970s, scientists have applied optics to determine the chemical composition of the human body. Noninvasive optical detection technologies include a variety of methods, such as near-infrared spectroscopy [11, 12], infrared spectroscopy [13], polarimetry [14], photoacoustics [15], Raman spectroscopy [16], and light-scattering coefficient method [17].

Near-infrared light, the wavelength of which varies from 780 nm to 2526 nm, is the electromagnetic wave between visible light and mid-infrared light that can penetrate the human skin and tissues. A good linear correlation exists between blood glucose concentrations and near-infrared spectrum absorption. In recent years, near-infrared spectroscopy measurement has been widely employed and has thus become fast-developing technology for analysis, particularly in medical applications [11, 18, 19]. The research into near-infrared spectroscopy combined with chemometrics is regarded as an effective method for the noninvasive detection of blood glucose concentrations [11, 20].

Empirical mode decomposition (EMD), which is an adaptive time frequency data analysis method, is widely used in nonsteady and nonlinear systems [21]. However, mode mixing occurs in EMD. For example, different oscillations exist in the same intrinsic mode function (IMF), or similar oscillations exist in different IMFs. This problem is addressed with ensemble empirical mode decomposition (EEMD), which employs EMD to integrated signals with white Gaussian noise [22]. However, signals with added noise can produce varying numbers of IMFs, and reconstructed signals contain residual noise after decomposition. In complementary ensemble empirical mode decomposition (CEEMD), which can completely eliminate the residual noise in reconstructed signals [23], pairs of positive and negative noises are added to a signal to improve the efficiency of the original noise auxiliary method. EEMD or CEEMD will produce wrong ingredients components, and the IMFs obtained via decomposition may fail to meet the definition of IMF when parameter selection is ineffective. These limitations are resolved with another noise auxiliary algorithm, called CEEMDAN, which is used to achieve an accurate reconstruction of original signals and pure decomposed mode spectra [24]. The iterations of CEEMDAN are less than half of the iterations of EEMD. Moreover, CEEMDAN can accurately reconstruct original signals and recover the features of EMD that are lacking in EEMD. However, CEEMDAN still has some problems which need to be improved; for example, its modes contain some residual noise, and the signal information shows some spurious modes in the early stages of decomposition [25]. To overcome these two issues, the improved CEEMDAN method is applied in this paper to obtain modes with less noise and more physical meaning.

The EMD-based methods can decompose the signal into a series of IMFs which contain the noisy modes and information modes. Therefore, it can be powerful adaptive tool to extract the sensitive intrinsic mode functions to reconstruct the signal. The problems is how to select the sensitive mode to distinguish relevant IMFs and irrelevant IMFs in an efficient way. Reference [26] uses an analogue approach based on consecutive mean squared error (CMSE) criterion. The signal is reconstructed from the mode whose criterion is minimal. In [27], the authors propose an intuitive selected mode method by a new criterion based on Hausdorff distance (HD). Moreover, [28] introduces the mutual information (MI) to select the sensitive IMFs which can reflect the signal characteristics for signal reconstruction. In this paper, the correlative coefficient is used to select relevant IMFs to extract useful spectral information.

Chemometrics, which was proposed by Bhattacharjee in 1994 [29], employs a multivariate statistical analysis of calibration methods and computing technologies to calculate the sample content of each component combined with the near-infrared spectrum. Common linear chemometrics modeling methods include multiple linear regression, principal components regression, and partial least squares (PLS) regression. Examples of nonlinear modeling methods include artificial neural networks and support vector regression (SVR). Generally, modeling is the process of selecting parameters and methods. SVR can obtain the global optimal solution in spectrum detection and convert linear regression to nonlinear regression, as well as kernel function to the linear mapping of high-dimensional space. The basic principle of SVR, which is a regression method developed from support vector machine, is to map the original data to high-dimensional feature space through nonlinear mapping and to establish a regression model in this space. Applying SVR in near-infrared spectrum quantitative analysis modeling produces a good effect. The commonly used kernel functions include linear kernel functions, polynomial kernel functions, radial basis kernel functions, and sigmoid kernel functions. Many researches and experiments demonstrate that radial basis kernel functions are preferable options if previous knowledge is insufficient. The particle swarm optimization- (PSO-) SVR method is proposed to select *ε*, *C*, and *γ* simultaneously. The results show the satisfactory learning precision and generalization ability with PSO-SVR.

The paper is organized as follows.  Section 2 provides a description of the spectrum reconstruction method based on improved CEEMDAN and the PSO-SVR model. The CEEMDAN algorithm, improved CEEMDAN algorithm, correlative coefficient, PSO, and SVR are also introduced.  Section 3 presents the near-infrared spectrum experiments on glucose solutions and the results of different modeling methods.  Section 4 presents the conclusion of the study.

## 2. Methods

### 2.1. CEEMDAN Algorithm

The basis of CEEMDAN is EEMD. Thus, the decomposition theory of the EEMD method is described first [24].

(1) Set *x* ^*i*^(*n*) = *x*(*n*) + *w* ^*i*^(*n*), where *w* ^*i*^(*n*)  (*i* = 1,…, *I*) is a different white Gaussian noise.
(2) The modes IMF_*k*_^*i*^(*n*) of each *x* ^*i*^(*n*)  (*i* = 1,…, *I*) can be obtained by EMD, where *k* = 1,…, *K* representatives modes.
(3) The *k*th mode of *x*(*n*) is set to ${\overset{-}{IMF}}_{k}$, and the corresponding average of IMF_*k*_^*i*^ is
  (1) $$\begin{array}{c} {\overset{-}{IMF}}_{k}\left(\;n\;\right)=\frac{1}{I}\overset{I}{\underset{i=1}{\sum }}{IMF}_{k}^{i}\left(\;n\;\right). \end{array}$$

In EEMD, each independently decomposed *x* ^*i*^(*n*) produces residue *r*_*k*_^*i*^(*n*) = *r*_*k*−1_^*i*^(*n*) − IMF_*k*_^*i*^(*n*). However, the decomposition modes are called ${\tilde{IMF}}_{k}$, and the first residue is $r_{1}\left(n\right)=x\left(n\right)-{\tilde{IMF}}_{1}(n)$, where ${\tilde{IMF}}_{1}(n)$ is obtained by employing EEMD. ${\tilde{IMF}}_{2}\left(n\right)$ is the mean value of the result. *r*_1_(*n*) with a different given noise is decomposed by EMD. The next residue is $r_{2}\left(n\right)=r_{1}\left(n\right)-{\tilde{IMF}}_{2}(n)$. Other modes continue this process until the stop condition is met.

Operator *E*_*j*_(·) is the *j*th mode of a given signal decomposed by EMD, where *w* ^*i*^ is the white noise with the mean value of zero and the variance of one.

If *x*(*n*) is the signal, then the steps of CEEMDAN are described as follows:

(1) The signal *x*(*n*) + *ε*_0_ *w* ^*i*^(*n*) is decomposed by EMD *I* times to obtain the first mode: ${\tilde{IMF}}_{1}\left(n\right)=(1/I)\sum_{i=1}^{I}{IMF}_{1}^{i}\left(n\right)={\overset{-}{IMF}}_{1}(n)$.
(2) When *k* = 1, the first residue $r_{1}\left(n\right)=x\left(n\right)-{\tilde{IMF}}_{1}(n)$ is calculated.
(3) *r*_1_(*n*) + *ε*_1_ *E*_1_(*w* ^*i*^(*n*))  (*i* = 1,…, *I*) is decomposed until the first EMD mode is obtained. The second mode is then calculated:
  (2) $$\begin{array}{c} {\tilde{IMF}}_{2}\left(\;n\;\right)=\frac{1}{I}\overset{I}{\underset{i=1}{\sum }}E_{1}\left(\;r_{1}\left(\;n\;\right)+\varepsilon_{1}E_{1}\left(\;w^{i}\left(\;n\;\right)\;\right)\;\right). \end{array}$$
(4) When *k* = 2,…, *K*, the *k*th residue $r_{k}\left(n\right)=r_{k-1}\left(n\right)-{\tilde{IMF}}_{k}(n)$ is calculated.
(5) *r*_*k*_(*n*) + *ε*_*k*_ *E*_*k*_(*w* ^*i*^(*n*))  (*i* = 1,…, *I*) is decomposed until the first EMD mode is obtained. The (*k* + 1)th mode is then defined:
  (3) $$\begin{array}{c} {\tilde{IMF}}_{\left(k+1\right)}\left(\;n\;\right)=\frac{1}{I}\overset{I}{\underset{i=1}{\sum }}E_{1}\left(\;r_{k}\left(\;n\;\right)+\varepsilon_{k}E_{k}\left(\;w^{i}\left(\;n\;\right)\;\right)\;\right). \end{array}$$
(6) Steps (4)–(6) are repeated until the obtained residue can not be decomposed; that is, the residue has a maximum of one extreme at most. The final residue meets $R\left(n\right)=x\left(n\right)-\sum_{k=1}^{K}{\tilde{IMF}}_{k}$, where *K* is the total mode number. Thus the expression of signal *x*(*n*) is
  (4) $$\begin{array}{c} x\left(\;n\;\right)=\overset{K}{\underset{k=1}{\sum }}{\tilde{IMF}}_{k}+R\left(\;n\;\right). \end{array}$$

### 2.2. Improved CEEMDAN Algorithm

According to [25], the improved CEEMDAN algorithm is described based on CEEMDAN as follows:

(1) For *x* ^*i*^(*n*) = *x*(*n*) + *β*_0_ *E*_1_(*w* ^*i*^(*n*)), calculate the local means of *I* realizations by EMD to obtain the first residue, *r*_1_ = 〈*M*(*x* ^*i*^(*n*))〉, where *β*_0_ = *ε*_0_std(*x*)/std(*E*_1_(*w* ^*i*^(*n*))) and *M*(·) is the operation which produces the local mean of the signal.
(2) When *k* = 1, calculate the first mode: ${\tilde{IMF}}_{1}\left(n\right)=x(n)-r_{1}$.
(3) Estimate the second residue as the average of local means of the realizations *r*_1_(*n*) + *β*_1_ *E*_2_(*w* ^*i*^(*n*)); then the second mode is defined
  (5) $$\begin{array}{c} {\tilde{IMF}}_{2}\left(\;n\;\right)=r_{1}-r_{2}=r_{1}-\langle \;M\left(\;r_{1}+\beta_{1}E_{2}\left(\;w^{i}\left(\;n\;\right)\;\right)\;\right)\;\rangle . \end{array}$$
(4) Calculate the *k*th residue and *k*th mode (*k* = 3,…, *K*):
  (6) r1=Mrk−1+βk−1Ekwin,
  (7) IMF~kn=rk−1−rk.
(5) Repeat (4) for the next *k*.

### 2.3. Correlative Coefficient

The correlative coefficient is widely applied in almost all areas of science and technology. The correlative coefficient is a dimensionless index used in multivariate statistics to represent the statistical relationship between two groups of variables. Its value ranges from −1 to 1, and it is divided into three classes, namely, positive correlation, irrelevant correlation, and negative correlation. Generally, certain processing in the computation is necessary to combine the negative correlation with the positive correlation. The value of the correlative coefficient ranges from 0 to 1, and a high value indicates a strong correlation. After setting the two groups of variables, namely, *x*, *y*, the correlative coefficient *ρ*_*xy*_ is

(8) $$\begin{array}{c} \rho_{xy}=\frac{cov\left(x,y\right)}{\sqrt{cov\left(x,x\right)\cdot cov\left(y,y\right)}}, \end{array}$$

where $cov\left(x,y\right)=(1/\left(n-1\right))\sum_{i=1}^{n}\left(x_{i}-\overset{-}{x})(y_{i}-\overset{-}{y}\right)$, $cov\left(x,x\right)=(1/\left(n-1\right))\sum_{i=1}^{n}{\left(x_{i}-\overset{-}{x}\right)}^{2}$, $cov\left(y,y\right)=(1/\left(n-1\right))\sum_{i=1}^{n}{\left(y_{i}-\overset{-}{y}\right)}^{2}$, $\overset{-}{x}=(1/n)\sum_{i=1}^{n}x_{i}$, and $\overset{-}{y}=(1/n)\sum_{i=1}^{n}y_{i}$.

Thus, the correlative coefficient can be expressed as

(9) $$\begin{array}{c} \rho_{xy}=\frac{\sum_{i=1}^{n}\left(x_{i}-\overset{-}{x}\right)\left(y_{i}-\overset{-}{y}\right)}{\sqrt{\sum_{i=1}^{n}{\left(x_{i}-\overset{-}{x}\right)}^{2}\cdot \sum_{i=1}^{n}{\left(y_{i}-\overset{-}{y}\right)}^{2}}}. \end{array}$$

### 2.4. Signal Reconstitution Method

Signal characteristics are not evident because of the overlapped hydrogen absorption peaks in the near-infrared spectrum. Moreover, the modeling result using the original spectroscopy data is inferior, and the accuracy is not high. Therefore, removing useless components can produce satisfactory predictions and simplifies the model. According to improved CEEMDAN and the correlation coefficient, the signal reconstitution method can be concluded by employing the following steps.

(1) The original signal is decomposed into IMF_*i*_  (*i* = 1,2,…, *n*) by using the improved CEEMDAN algorithm, and *n* is the number of IMFs.
(2) All the correlative coefficient value between IMF_*i*_ and the original signal is calculated using formula (9). The sensitive IMFs are selected according to the correlative coefficient threshold [30], which is shown in formula (10).
  (10) $$\begin{array}{c} \mu_{h}=\frac{\max\left(\mu_{i}\right)}{10\times \max\left(\mu_{i}\right)-3}\;\left(i=1,2,\ldots ,n\right). \end{array}$$
 In the formula above, *μ*_*i*_ represents the correlative coefficient between IMF_*i*_ and the original signal, and the maximum number of correlative coefficient is denoted by max⁡(*μ*_*i*_).
 If the correlative coefficient value between IMF_*i*_ and the original signal is larger than *μ*_*h*_, then the relevant IMF is maintained as the sensitive mode. Otherwise, the relevant IMF is removed as a false component.
(3) The sensitive IMFs are selected to reconstruct the signal for modeling.

### 2.5. PSO-SVR Modeling Method

The PSO algorithm is a type of parallel global search strategy that is based on population. It is easy to implement, and its concept is relatively simple; in PSO, many parameters no longer require adjustments. PSO exhibits a fast convergence speed and the capability of dealing with high-dimensional problems.

The speed-position model is used in the PSO algorithm. In the *D*-dimension solution space, the position of the *i*th particle in the group is *X*_*i*_ = (*x*_*i*1_, *x*_*i*2_,…,*x*_*iD*_)^*T*^, and the velocity ratio is *V*_*i*_ = (*v*_*i*1_, *v*_*i*2_,…,*v*_*iD*_)^*T*^. The individual extreme value at the current time is *p*_*ibest*_, and the global extreme value is *g*_*best*_. In each iteration process, the particles adjust the position and velocity of the current time by tracking the individual extreme value and global extreme value and state in the previous time. The iterative formula is shown as follows:

(11) vik+1=w∗vik+c1∗rand⁡ ∗pibest−xik+c2∗rand⁡ ∗gbest−xik,Xik+1=Xik+Vik+1,

where *V*(*k*), *V*(*k* + 1), *X*(*k*), *X*(*k* + 1) are the velocity and position at the current moment and next moment, respectively; rand ⁡( ) is the random number within [0, 1], and *c*_1_, *c*_2_ are the learning factors which are usually equal to two. *ω* is the weighting factor that should automatically decrease with algorithm iteration to accelerate convergence speed; it is generally defined as

(12) $$\begin{array}{c} \omega =\omega_{min}+\frac{\left({iter}_{max}-iter\right)*\left(\omega_{max}-\omega_{min}\right)}{{iter}_{max}}, \end{array}$$

where *ω*_max_ and *ω*_min_ are the maximum and minimum weighting factors, respectively, iter is the current iteration number, and iter_max_ is the total iteration number.

For the sample data set {*x*_*i*_, *y*_*i*_}  (*i* = 1,2,…, *n*, *x*_*i*_ ∈ *R* ^*d*^, *y*_*i*_ ∈ *R*), the regression function obtained by SVR fitting is

(13) fxw·∅x+b=∑i=1na^−aiφxi·φx+b∗=∑i=1na^−aikxi,x+b∗,

where *a*_*i*_ and $\hat{a}$ are Lagrangian operators and *b* ^*∗*^ is the threshold. Consider the following:

(14) Kxi,yi=exp⁡−xi−xj22γ2  is  the  kernel  function.

For PSO-SVR, the position and velocity of each particle are determined by 3D parameters (*ε*, *C*, *γ*). The mean square error (MSE), which can directly reflect the regression performance of SVR, is used as the fitness function:

(15) $$\begin{array}{c} MSE={\left(\;\sum_{i=1}^{n}\frac{\left(\hat{y}-y_{i}\right)}{n}\;\right)}^{1/2}, \end{array}$$

where $\hat{y}$ is the estimated value of a new sample.

The steps for the optimal selection of parameters (*ε*, *C*, *γ*) in PSO-SVR are described as follows.

(1) The particle swarm *ε*, *C*, *γ* are initialized. Group size *m* is determined, the maximum and minimum weighting factors of algorithms *ω*_max_, *ω*_min_ are identified, and the maximum iteration number iter_max_ is set.
(2) The individual extreme value *p*_*ibest*_ of each particle is set as the current position. The fitness of each particle is set using the fitness function, namely, formulas (13) and (15). The individual extreme value corresponds to the particle with the best fitness as the global extreme value *g*_*best*_.
(3) On the basis of steps (1)–(3) for iteration calculation, the position and speed of particle are updated.
(4) The fitness of each particle is evaluated using formulas (13) and (15).
(5) If the fitness of each particle is better than the corresponding fitness *p*_*ibest*_, then *p*_*ibest*_ is updated. Otherwise, the original value is retained.
(6) If the updated *p*_*ibest*_ of each particle is better than the global extreme value *g*_*best*_, the *g*_*best*_ is updated. Otherwise, the original value is retained.
(7) If the maximum iteration is reached or if the solution does not change, the iteration is stopped. Otherwise, the process returns to step (3).

## 3. Experimental Results and Discussion

### 3.1. Simulation Signal Reconstruction Experiments

**Figure 1:**
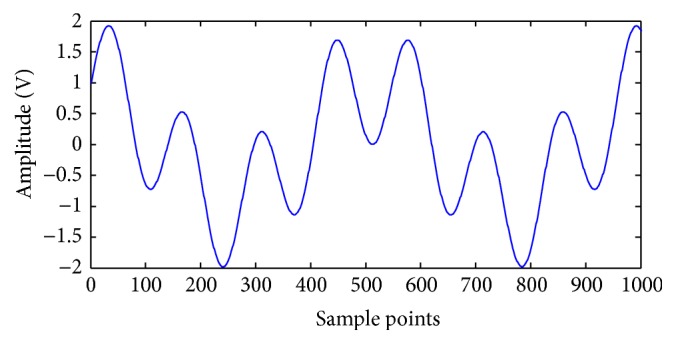
Waveform of pure signal.

**Figure 2:**
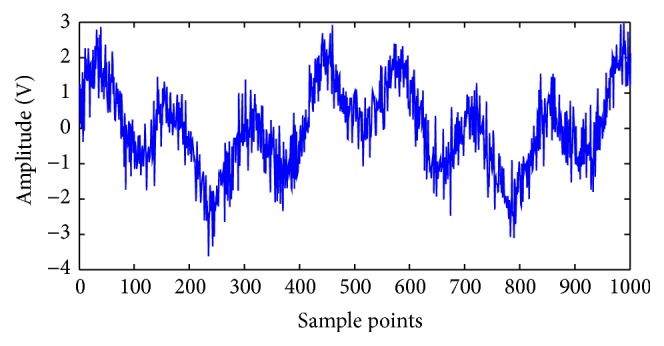
Waveform of noisy signal.

**Figure 3:**
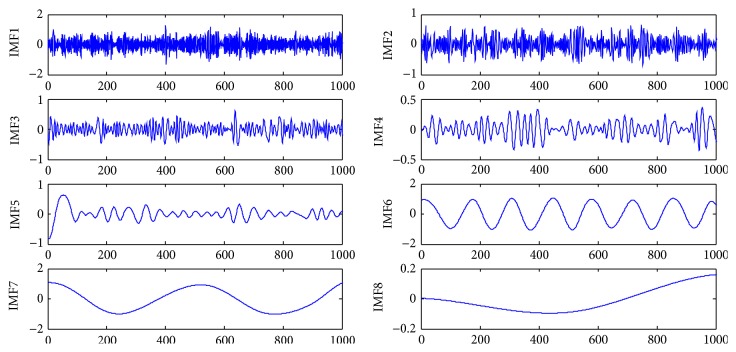
Waveform of IMFs.

**Figure 4:**
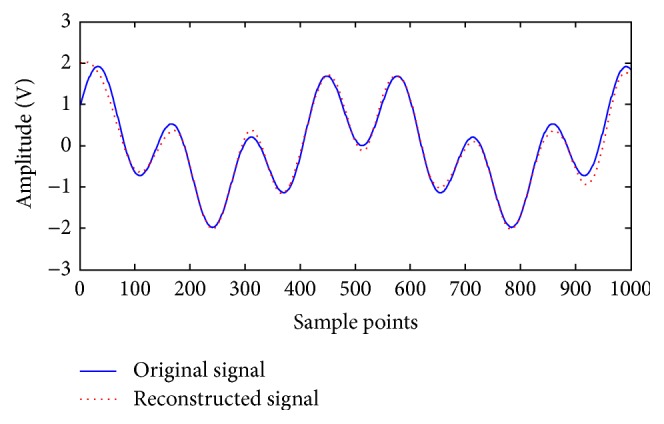
Waveform of reconstructed and original signal.

Consider the original signal *y*(*t*) = cos⁡ (4*πt*) + sin⁡ (15*πt*). The length of the data is 1024, as shown in Figure 1. White Gaussian noise is added to the original signal with the input signal to noise ratio (SNR) fixed at 5 dB. Noisy signal *y*(*t*) (Figure 2) is decomposed into eight modes.  Figure 3 indicates that the sixth and seventh modes are two components of a pure signal. In the proposed method, the correlative coefficients between each IMF and noisy signal *y*(*t*) are calculated, and the threshold is obtained with formula (7). The reconstructed signal is the sum of IMFs with correlative coefficients larger than the threshold. For noisy signal *y*(*t*), the correlative coefficients of IMF6 and IMF7 are larger than the threshold (0.16721), which is shown in Table 1. The IMFs are arranged from high to low frequency, and the noise is often concentrated around the first IMFs. The first three modes should be removed to reconstruct the signal regardless of whether the correlative coefficients are larger than the threshold in the proposed method. The reconstructed signal is presented in Figure 4.

The simulated signal *y*(*t*) is reconstructed to effectively reconstruct the spectral signal, and the reconstruction results for EMD, EEMD, CEEMD, CEEMDAN, and improved CEEMDAN are compared. In the experiments, the ensemble number and white Gaussian noise are fixed (the ensemble number is 100, and the SNR of white noise is 5 dB). The reconstruction effect is evaluated by introducing the SNR, root mean square error (RMSE), and correlative coefficient into this method. The SNR and RMSE are defined as

**Figure 5:**
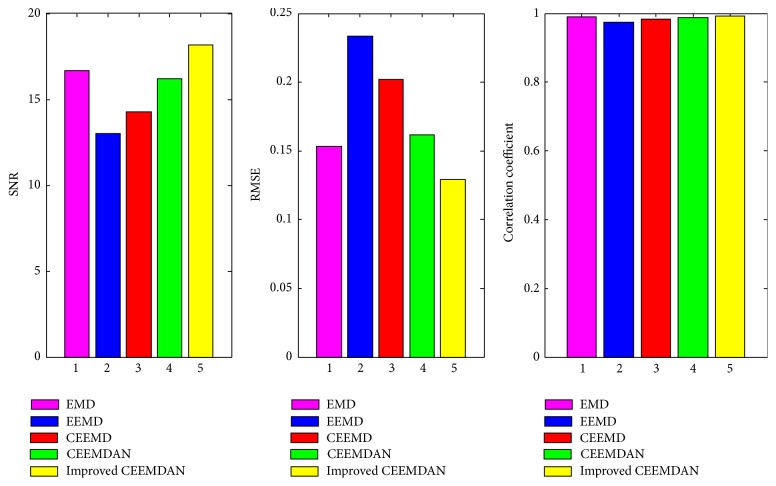
SNR, RMSE, and correlative coefficient for *y*(*t*).

(16) SNR=10 log10⁡∑t=1Nyt2∑t=1Ny~t−yt2,RMSE=1N∑t=1Ny~t−yt2,

where $\tilde{y}\left(t\right)$ is the reconstructed signal and *y*(*t*) is the original signal.  Figure 5 demonstrates that EMD and improved CEEMDAN produce high SNR and small RMSE values. Hence, the reconstruction errors of these methods are smaller. However, EMD exhibits serious mode mixing. Moreover, the correlative coefficient values of these five methods are approximate, and the reconstruction effect can not be identified (Figure 5). Combining the SNR, RMSE, and correlative coefficients results (Table 2), the improved CEEMDAN method with the correlative coefficient exhibits a strong robustness to signal reconstruction and can extract useful signal information.

The experiment results from Figure 5 and Table 2 show that improved CEEMDAN method is better than that of the EMD, EEMD, CEEMD, and CEEMDAN. It is more suitable for the decomposition of nonstationary signal. For the signal *y*(*t*), the proposed selected mode method based on correlative coefficient with improved CEEMDAN decomposition is compared with the methods in [26–28]. By the results shown as in Table 3, the correlative coefficient values of these four methods are approximate; however, the reconstruction effects of proposed method whose SNR is 18.2289 and RMSE is 0.1289 are superior to that of the other three methods. Therefore, the proposed method based on improved CEEMDAN has small reconstruction error and strong denoising ability.

### 3.2. Near-Infrared Spectrum Experiments

#### 3.2.1. Near-Infrared Spectrum of the Glucose Solution

**Figure 6:**
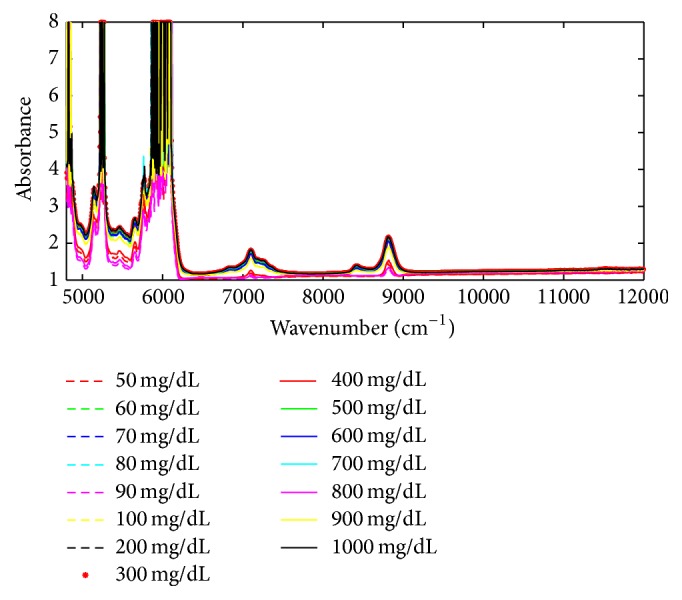
Some of the glucose solution spectrum.

The instrument utilized for near-infrared spectroscopy is the Antaris II FT-NIR, produced by America Thermo Company. It is used to carry out full spectrum scanning transmission in the spectrum ranging from 833 nm to 2630 nm. The near-infrared spectroscopy of the 75 samples of 15 groups with different concentrations (range of 50–1000 mg/dL) of glucose solutions is selected to establish the spectrum calibration model. In the actual experiments, the transmission spectroscopy of the same sample is carried out several times to avoid the influence of instability of the near-infrared spectroscopy and to improve accuracy. Thus, in the modeling process, the average signal obtained from five measuring times for one solution sample is considered the training data. Some of the glucose solution spectra data are shown in Figure 6. The near-infrared spectrum of the samples is decomposed and reconstructed by employing improved CEEMDAN, which can eliminate the noise caused by some uncertain factors, such as spectrometer accuracy or test conditions, and can accurately provide spectral information for the calibration model.

#### 3.2.2. Near-Infrared Spectrum Calibration Model Building

To maximize the sample data, we use cross validation until all the samples are tested once. The sample spectrum is collected independently. Thus, any individual can be chosen as the testing sample, and others are considered as training samples to evaluate the modeling method. PSO-SVR only needs a small number of selected samples for training, particularly the data sets that are sensitive to noise, and can thus reflect the advantages of SVR and the intelligent optimization algorithm. To realize PSO-SVR, the constitution and prediction algorithms of SVR are embedded in the steps to calculate the fitness value in the PSO algorithm. Reference [31] provides the approximate scope of *ε*, *C*, *γ* as *ε* = [0,0.2], *C* = [1, 10^8^], and *γ* = [0.01,2]; *ω*_max_, *ω*_min_, and *m* are generally taken as 0.9, 0.4, and 10, respectively. These initialization parameters can avoid choice blindness.

PLS regression, a classical calibration method, can extract the latent variables associated with the dependent variable in the spectrum and establish the regression equation. In this work, the PLS is the comparison method for PSO-SVR. PSO-SVR and PLS are utilized to establish the calibration model between the spectral data and the real glucose concentration.

After establishing the calibration model, the results must be verified to confirm the reliability of the model. The correlation coefficient and the root mean square error of prediction (RMSEP) are employed to evaluate the correlation of the model. The correlation coefficient can describe the linear correlation degree between the spectral matrix *X* and the concentration matrix *Y*; it is commonly denoted by *R*. when the *R* value is closer to one, the regression effect of model is satisfactory. Moreover, when the RMSEP is small, the prediction precision of model is high. The correlation coefficient and RMSEP are calculated as

(17) R=1−∑y^i−yi2∑y^−i−yi2,RMSEP=∑y^i−yi2n−1,

where *n* is the sample quantity of the calibration set, *y*_*i*_ is the true value of the *i*th sample, ${\hat{y}}_{i}$ is the predicted value of the *i*th sample, and ${\overset{-}{\hat{y}}}_{i}$ is the average value of ${\hat{y}}_{i}$ of all the samples in the calibration set.

**Figure 7:**
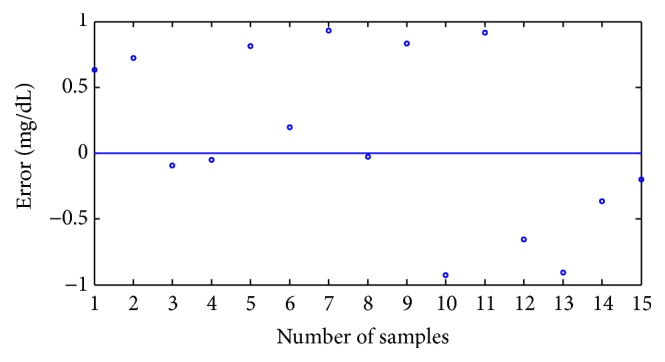
The errors between predicted values and true values.

**Figure 8:**
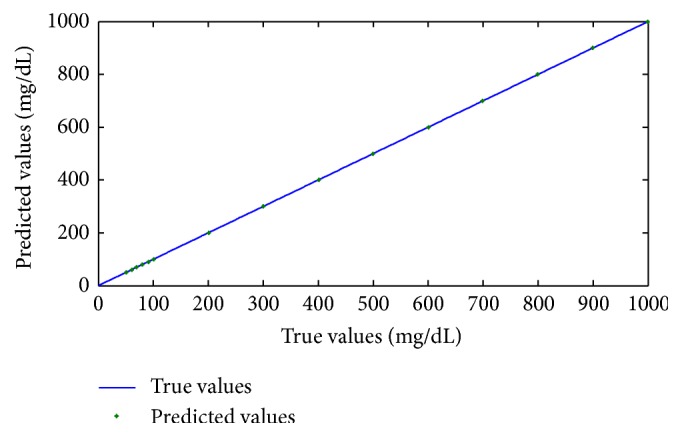
The predicted values and true values of cross validation.

Two types of calibration models introduced above are employed to predict the glucose concentrations in 15 glucose solutions samples. The errors between the predicted values and the true values are then calculated. The results are shown in Figure 7. The predicted values and true values are provided in Figure 8.  Table 4 shows that the PSO-SVR, which is based on improved CEEMDAN for predictions and whose *R* and RMSEP are 0.9999997 and 0.5352, respectively, is more robust and accurate than PLS, whose *R* and RMSEP are 0.9999825 and 0.91, respectively. The *R* and RMSEP values of the calibration model established with the spectrum data, which were reconstructed with improved CEEMDAN in the PLS and PSO-SVR methods, are better than those of the calibration model with the original spectrum data. Thus, PSO-SVR, which is based on improved CEEMDAN, exhibits a good performance in detecting the glucose concentrations. The excellent prediction results of PSO-SVR indicate that near-infrared spectroscopy technology can be employed to detect glucose concentrations.

## 4. Conclusion

The quantitative analysis of near-infrared spectroscopy data demonstrates the potential development in noninvasive blood glucose detection. PSO-SVR is an effective method to solve the regression problem on high-dimensional data matrixes. This study proposes the PSO-SVR modeling method that is based on the improved CEEMDAN algorithm. The proposed method can remove noise, extract useful information from near-infrared spectra, and optimize the parameters in SVR. The near-infrared spectrum analysis model established with the PSO-SVR based on improved CEEMDAN method is stable, accurate, and practicable and it exhibits a good predictive effect. The current experiments focus on glucose solutions. In the future, we will extend the experiments to human tissues.

## Figures and Tables

**Table 1:** Correlative coefficients between each mode and noisy signal.

Mode	IMF1	IMF2	IMF3	IMF4	IMF5	IMF6	IMF7	IMF8
Correlative coefficient	−0.0125	−0.0096	0.0054	0.0073	0.0579	**0.7215**	**0.7429**	0.0592

**Table 2:** Values of SNR, RMSE, and correlative coefficient for reconstruction signal.

Methods	SNR	RMSE	Correlative coefficient
EMD	16.6707	0.1533	0.9896
EEMD	13.0117	0.2336	0.9754
CEEMD	14.2667	0.2021	0.9824
CEEMDAN	16.2156	0.1615	0.9888
Improved CEEMDAN	18.1517	0.1292	0.9924

**Table 3:** Values of SNR, RMSE, and correlative coefficient for different methods.

Methods	SNR	RMSE	Correlative coefficient
Improved CEEMDAN-CMSE	13.1254	0.2305	0.9817
Improved CEEMDAN-HD	16.8698	0.1498	0.9914
Improved CEEMDAN-MI	14.4521	0.1979	0.9835
Proposed method	18.2289	0.1289	0.9952

**Table 4:** Values of *R* and RMSEP for calibration model.

Methods	*R*	RMSEP
PLS	0.9999825	0.9100
Improved CEEMDAN-PLS	0.9999985	0.6519
PSO-SVR	0.9999986	0.5560
Improved CEEMDAN-PSO-SVR	0.9999997	0.5352
